# Community Characteristics, Victimization, and Psychological Adjustment Among School-Aged Adopted Children With Lesbian, Gay, and Heterosexual Parents

**DOI:** 10.3389/fpsyg.2020.00372

**Published:** 2020-03-10

**Authors:** Abbie E. Goldberg, Randi Garcia

**Affiliations:** ^1^Department of Psychology, Clark University, Worcester, MA, United States; ^2^Smith College, Northampton, MA, United States

**Keywords:** adopted, bullying, gay, lesbian, psychological adjustment, same-sex, school-aged, victimization

## Abstract

Little research has examined victimization among school-aged children raised in lesbian/gay (LG) parent households and almost no work has attended to the school and community contexts that may impact their victimization risk. This study examined predictors of parent-reported child victimization and child adjustment, and parent responses to victimization, in 43 two-mother, 37 two-father, and 56 mother–father families, with adopted children (median age = 8.6 years). Predictors included parent (sexual orientation), school (climate, public versus private) and community (urbanicity, percentage voted Democrat) factors, with parent and child demographics included as controls. A total of 47% of parents reported one or more child victimization experiences in the past year; there were no differences by family type. An exploratory interaction between family type and urbanicity indicated that in large urban areas, children with LG parents were predicted to experience less victimization than children with heterosexual parents; in more rural regions, children with LG parents were predicted to experience more victimization than children with heterosexual parents. School climate was related to victimization: Parents who reported more negative school climate reported more child victimization. Children with higher levels of parent-reported victimization had higher levels of parent-reported internalizing and externalizing symptoms. In large urban areas, children with LG parents were predicted to have fewer internalizing symptoms than children with heterosexual parents; in more rural areas, children with LG parents were predicted to have more internalizing symptoms than children with heterosexual parents. Regarding parents’ responses to victimization, LG parents were more likely to talk to school administrators, their children, and the bully, compared to heterosexual parents.

## Introduction

Over the past two decades, attitudes about lesbian and gay (LG) couples becoming parents have become more positive (Daugherty and Copen, 2016), although stigmas remain (Ioverno et al., 2018), such that some LG couples who seek to become parents still face hostility from reproductive (Wingo et al., 2018) and adoption (Goldberg et al., 2019) services. Despite such challenges, LG couples are increasingly becoming parents, particularly through adoption (Goldberg, 2010). In turn, LG-parent families are now part of the social fabric of the communities in which they reside, including their neighborhoods and schools. Although research has gradually begun to address the experiences of LG-parent families (Goldberg, 2010), rarely has it considered their intersections with the school context. Studies of LG parents of young children have examined their school decisions (Goldberg et al., 2018) and school involvement (Goldberg and Smith, 2014; Goldberg et al., 2017) and several studies of LG-parent families with school-aged children have explored children’s experiences with teasing and victimization (Bos and van Balen, 2008; Kosciw and Diaz, 2008; Rivers et al., 2008; Farr et al., 2016).

The current study aims to examine the role of child, family, school, and community factors in predicting LG^1^ and heterosexual parents’ reports of their school-aged adopted children’s experiences of victimization, including overtly aggressive behaviors such as physical or verbal aggression (direct victimization) and behaviors such as rumor spreading, exclusion, and ignoring (indirect victimization). Our study is informed by an ecological perspective (Bronfenbrenner, 1988), which orients us to consider the family, school, and broader community contexts, and their intersections, in shaping children’s development (Beveridge, 2005). For example, children are impacted directly by their family and school—two proximal contexts—as well as by distal contexts, such as local and state norms, policies, and laws, all of which may impact the child directly and indirectly (e.g., via their influence on school policies and practices).

Children whose parents are LG—and who are also adopted—may experience unique risk factors for victimization. As children enter middle childhood, they develop a greater awareness of what it means to have LG parents (Goldberg, 2010) and a greater sense of their adoptive identities (Brodzinsky, 2011) and may be increasingly vulnerable to stigma regarding these personal and family identities. Middle childhood is a developmental period marked by increased independence from parents and more time with peers, and is characterized by new challenges in navigating social hierarchies (Merrin et al., 2018). By extension, bullying in general and homophobic teasing specifically tend to peak in middle school (Merrin et al., 2018). In turn, some work suggests that elementary and middle school represent periods of more intense homophobic teasing for youth with LG parents (Kosciw and Diaz, 2008), while other work suggests that the number of children being teased for having LG parents is relatively low (8% in one study using parent reports for school-aged adopted youth; Farr et al., 2016). Unknown are what school and community factors are related to child victimization in LG-parent families, and how these interact with the family context to predict victimization.

Using a sample of 136 families (43 two-mother families, 37 two-father families, and 56 mother–father families, with adopted children; median age = 8.6), the main question this study seeks to answer is: What parent factors (e.g., sexual orientation), child factors (e.g., race, gender), school factors (e.g., school climate; public vs. private) and community factors (e.g., urbanicity) predict parents’ perceptions of their children’s victimization? Two exploratory subquestions are: Are these factors related to *children’s* reports of victimization? Is parent sexual orientation related to *parents’ responses* to victimization? A second question we seek to answer is whether victimization is related to child psychological adjustment.

### Family Structure as a Predictor of Victimization

Of interest is whether family structure matters in terms of predicting victimization: that is, whether LG parents of school-aged children report greater levels of victimization in their children than heterosexual parents. Research generally finds that overall levels of victimization may not differ between groups, but suggests that there may be differences in the nature of responses to victimization. Rivers et al. (2008) studied 18 youth ages 12–16 who were raised in same-sex parent families and compared them to a matched sample of students raised in different-sex parent families and found no differences in victimization between the two groups. Yet youth with same-sex parents were less likely to report that they would turn to school-based supports (e.g., school staff), which the authors hypothesized may reflect fears of encountering stigma from these sources. Similarly, Wainright and Patterson (2006) found that adolescents (12–18 years) in two-mother families reported no differences in victimization compared to adolescents in mother–father families, and Bos and van Balen (2008) reported low levels of teasing among 8- to 12-year-old children in two-mother families.

Overall levels of child victimization may not differ between LG- and heterosexual-parent families. Yet there is reason to believe that victimization might intersect with or vary according to where families live or the types of schools children attend—dimensions that have generally not been explored quantitatively. A qualitative study found that LG-parent families living in rural and politically conservative communities encountered unique challenges in school selection, amidst implicit and explicit biases against their families (Goldberg et al., 2018). Thus, it is worth considering community factors in exploring children’s exposure to victimization.

### Community Factors as Predictors of Victimization

Urbanicity is an important dimension of communities that may be related to victimization among youth with LG parents. Large cities tend to have more LGBTQ residents, as well as more services and resources for LGBTQ people, and are therefore often regarded as socially progressive and accepting of diversity (Holman and Oswald, 2011; Oswald and Holman, 2013). By contrast, LG-parent families in rural areas often lack access to LGBTQ inclusive services in health care, religious settings, schools, and other contexts, thereby reflecting and contributing to a more negative community climate (Oswald and Holman, 2013). Insomuch as urbanicity is often associated with a more LGBTQ-friendly community climate (Oswald and Holman, 2013; Williams Institute, 2016), it follows that LG-parent families in cities may experience their communities as more affirming (less hostile) than those in more rural areas.

One study documented a link between urbanicity and victimization among children with LGBTQ parents. Power et al. (2014) studied 455 Australian LGBTQ parents of children of varying ages, and found that parents living in small or medium metro areas, and rural areas, were less likely than those in large metro areas (urban centers) to feel connected to their community, be “out” in community settings, and have contact with the LGBTQ community. According to parents, children in the former group were more likely to experience homophobic bullying at school, highlighting a potential relationship between community setting and victimization risk. This study is important, but limited in its reliance on a crude self-report measure of urbanicity, assessment of one type of bullying, use of one respondent report per family, and non-inclusion of heterosexual-parent families. Research on LGBTQ youth has found similar associations. A study of LGBTQ youth ages 14–18 documented greater levels of victimization among participants living in what they perceived as hostile and small towns (Paceley et al., 2017). And, research on LGBTQ youth (mean age = 15.9 years) found that youth living in rural communities reported higher levels of victimization (Kosciw et al., 2009).

The political affiliation or voting history of a region or county may also have implications for the social climate in which youth with LG parents live and attend school. Republicans overall are less accepting of homosexuality, gay adoption, and marriage equality, compared to Democrats (Pew Research Center, 2017), and countywide support for the Republican party is associated with support for gay marriage bans (Burnett and Salka, 2009). In turn, even amidst advancements in marriage and parenthood legislation, sexual minorities who reside in more conservative counties report lower social inclusion and belongingness (Metheny and Stephenson, 2018) and poorer health (Hatzenbuehler et al., 2017) than those in more progressive areas. Amidst evidence that residents of the Southern and Midwestern regions of the United States report less tolerant attitudes toward LGBTQ people than those in other regions (Baunach, 2012), it is perhaps unsurprising that LGBTQ youth living in the South and Midwest were found to report marginally higher levels of victimization in school related to their gender expression compared to LGBTQ youth in the Northeast (Kosciw et al., 2009), highlighting how schools may reflect (i.e., be infused by) the norms and attitudes of the regions in which they are located.

### School Factors as Predictors of Victimization

Aspects of schools, which are embedded in communities, likely impact the nature, frequency, and targets of peer victimization. School climate—the overall or shared quality of school life—typically encompasses different aspects of the school environment, including social aspects (e.g., quality of teacher-student relationships), safety, and/or academic dimensions (e.g., emphasis on academic attainment) (Muijs, 2017). School climate is a key factor in promoting positive emotional, behavioral, and academic outcomes (Hendron and Kearney, 2016) and reducing negative risk factors (Thapa et al., 2013), including bullying (Bradshaw et al., 2009; Cook et al., 2010). For example, Attar-Schwartz (2009) studied 7th–11th graders and found that students with more negative perceptions of school climate were also more likely to report being victimized. Although school climate is most often measured via student and teacher reports, parent perceptions represent an arguably important viewpoint with regard to the school environment, especially in studies of younger children, who may be limited in their ability to provide reliable reports (Schueler et al., 2014).

Another factor that may relate to children’s victimization risk is school type. Private schools and public schools tend to differ in terms of the nature of school governance, as well as class size and teacher to student ratio (higher for public, lower for private; National Center for Education Statistics, 2019). In turn, private schools may, on average, be safer and healthier learning environments, and be associated with a reduced risk for victimization (Brinig and Garnett, 2012; Henkel and Slate, 2013). Research has generally found higher overall levels of bullying in public schools than private schools (Shujja et al., 2014; Waasdorp T. et al., 2018), although higher levels of cyberbullying specifically have been documented in private schools (Mark and Ratliffe, 2011; Waasdorp T. et al., 2018).

### Child Demographic Characteristics as Predictors of Victimization

Card et al. (2008) consider risk for victimization within an ecological context, whereby they outline features of the child’s context as well as personal characteristics that operate as risk and protective factors. In turn, both social-ecological and individual (person-level) correlates of victimization risk have been identified in the literature.

Children’s personal characteristics (e.g., race, gender, age) are often examined in relation to victimization risk. Some scholars suggest that victimization risk is related to stigmatized characteristics or perceived group affiliations such as race or ethnicity (Garnett et al., 2014), yet such findings are mixed (Tippett et al., 2013), with some studies finding lower levels of victimization among youth of color, compared to White youth (Lleras, 2008), and others finding higher levels (Goldweber et al., 2013). Some work has found race to be unrelated to victimization (Morrow et al., 2014).

Regarding child gender, some work suggests higher levels of overall victimization in boys than girls, among middle schoolers (Cook et al., 2010). Other research on elementary and middle school students shows higher levels of indirect victimization among girls (Waasdorp et al., 2011) and higher levels of direct victimization among boys (Waasdorp et al., 2011). Other work has found few gender differences in victimization (Morrow et al., 2014).

Finally, child age may also predict victimization. Victimization rates appear to rise from elementary school to middle school, peaking in middle school as children enter adolescence, and then tend to decline in high school (Cook et al., 2010; Espelage et al., 2018).

Little work has explored victimization among adopted children in general, although some research suggests more peer problems and lower psychosocial functioning among adopted children compared to non-adopted children (Pitula et al., 2019). A study of 9- to 15-year-old children adopted in the United States from Finland found that 19% reported being bullied, with boys being more likely to be victimized than girls (Raaska et al., 2012). A study of 5- to 13-year-old adopted children in the United Kingdom found that over half reported uncomfortable questions or teasing from peers about adoption specifically (Neil, 2012). Given the paucity of work on victimization among adopted children, our study makes a contribution to this literature as well.

### Predictors of Parents’ Responses to Victimization

Although much of the research on victimization has focused on children’s experiences, there is growing attention to the role of parents with regard to how they respond to and help their children cope with victimization. Parents may respond to victimization by contacting the school, talking to their child, or talking to the perpetrator’s parents (Waasdorp et al., 2011; Larrañaga et al., 2018; Lindstrom et al., 2019). Research with parents of victimized youth in elementary, middle, and high school suggests that most parents respond by talking to their child about victimization and/or contacting the school (Waasdorp et al., 2011; Lindstrom et al., 2019). Less frequently endorsed responses include talking to the bully’s parents and controlling the child’s internet access (Larrañaga et al., 2018). Although little work has examined the question of which of these represent the most ideal or effective responses, qualitative research suggests that children worry that parents contacting the bully or the bully’s parents may make the bullying worse (Mishna et al., 2006).

School, child, and family factors may impact parents’ responses to victimization. Some work shows that parents who view their child’s school climate more positively are less likely to respond by contacting the school (Waasdorp et al., 2011; Lindstrom et al., 2019) or talking to their child (Waasdorp et al., 2011). Child age and gender may also impact parents’ responses. A study of parents of 7th–10th graders found that parents of younger children were more likely to contact a teacher or school staff member or control internet/cellphone use in response to victimization, whereas parents of older children were more likely to encourage them to defend themselves (Larrañaga et al., 2018). Parents of girls were more likely to tell their children to ignore the problem or do nothing than parents of boys (Larrañaga et al., 2018).

Parent sexual orientation may also influence parent responses to victimization. As noted, Rivers et al. (2008) found that bullied youth with LG parents were less likely to turn to school-based supports. Perhaps LG parents also experience less trust that schools (which are frequently heteronormative in their policies and practices, and employ staff who lack comfort with LG-parent families; Goldberg and Smith, 2014) will effectively support their families, and are less likely to turn to them if their child is mistreated. At the same time, research on LG parents of young children suggests that they are highly involved at school, in part because they hope that their proactive advocacy will facilitate more favorable treatment (Goldberg and Smith, 2014; Goldberg et al., 2017). In turn, LG parents may indeed turn to school-based supports amidst child victimization, especially if they are highly involved and therefore expect positive treatment.

### Victimization as a Predictor of Externalizing/Internalizing Problems

In addition to enhancing knowledge of what factors place youth at risk for victimization, it is also important to understand how victimization impacts their well-being. Scholars have documented negative psychosocial outcomes associated with experiences of victimization in elementary, middle, and high school youth (Bradshaw et al., 2015; Waasdorp T. E. et al., 2018). Victimization is consistently linked to internalizing problems such as depression (Cook et al., 2010; Cillessen and Lansu, 2015; Waasdorp T. et al., 2018) and has sometimes been linked to externalizing problems (Cillessen and Lansu, 2015).

A small body of work has explored these associations in children with LG parents. In their study of adopted children raised in LG-parent families (mean age = 8 years), Farr et al. (2016) found that bullied children exhibited more behavioral problems than non-bullied children. Using a sample of 10- to 12-year-old children in two-mother families, Bos and van Balen (2008) found that higher levels of stigmatization were related to more problem behavior and lower self-esteem. Bos and Gartrell (2010) studied adolescents (mean age = 17 years) in two-mother families and found that greater stigmatization was associated with more problem behavior.

### The Current Study

This study utilizes a sample of 136 same-sex and heterosexual couples (dyads) with school-aged adopted children to answer the following research questions:

1. What parent, child, school, and community factors predict parents’ perceptions of their children’s victimization experiences?
  a. Hypothesis: We expect that higher victimization will be reported among parents who report less positive school climate, whose children attend public schools, who live in rural areas, and who live in Republican leaning communities. We expect no association between parent sexual orientation and victimization.
  b. *Exploratory interaction:* Does parent sexual orientation interact with community context (i.e., urbanicity) to predict children’s victimization experiences?
  c. *Exploratory follow-up*: In a subsample of 80 children with available data, do these same factors predict children’s reports of victimization experiences?
  d. *Exploratory follow-up:* Is parent sexual orientation related to parents’ responses to victimization?
2. Are parents’ reports of victimization related to children’s psychological adjustment?
  a. Hypothesis: We expect that victimization will be related to adjustment, such that higher victimization will be associated with lower adjustment (more problems).

As we are primarily interested in the role of parents’ sexual orientation, the school context, and the community context, we consider these as substantive predictors. We consider child demographics (age, gender, race) and parent demographics (income, education) as controls.

## Materials and Methods

### Sample

The parents in this study were originally recruited through adoption agencies for a study on the transition to adoptive parenthood (Goldberg and Smith, 2013). Approximately 8 years post-adoption, they participated in a follow-up assessment focusing on their child’s transition to elementary school. A total of 136 families participated: 43 two-mother, 37 two-father, and 56 mother–father families, all with adopted children. Child age ranged from 8 to 16 years old with a median age of 8.6 years. A total of 67% of the children were children of color, and 52% were boys. The majority (73%) were attending public schools.

A total of 35% of families resided on the East Coast, 36.5% on the West Coast, 10.0% in the Midwest, and 18.5% in the South. About 47% of participants resided in large central metro areas (e.g., Chicago, IL, United States), 22% in large fringe metro areas (e.g., Austin, TX, United States), 20% in medium metro areas (e.g., Lancaster, PA, United States), 7.8% in small metro areas (e.g., Missoula, MT, United States), and 3% in micropolitan/non-core areas (e.g., Greenfield, MA, United States). Participants lived in relatively Democratic communities, such that examination of the voting records in participants’ counties revealed that 63% of community members on average had voted Democrat in the last presidential election (*SD* = 15.8%). The sample was somewhat more affluent than national samples of adoptive parents (e.g., annual income is about $10K higher; Gates et al., 2007). Family income ranged from $15K to $750K with a median of $134K. The sample was well-educated, with 57% having master’s degrees or higher, 30% up to a college degree, and 13% a high school diploma/GED or lower. See Table 1 for sample demographics by family structure. Same-sex parents adopted a greater percentage of children of color than heterosexual parents. In addition, they tended to have higher family incomes and to live in communities with a higher Democratic voting percentage than heterosexual parents.

**TABLE 1:** Demographics by family type.

	Family type		
	Heterosexual	Same-sex	
Variable	*M* (*SD*) or % (*n*)	*M* (*SD*) or % (*n*)	*t* or *χ^2^*
**Child variables**			
Child of color	58.76% (57)	73.61% (106)	5.18*
Child’s age	8.74 (1.32)	9.12 (1.78)	−1.89^+^
Preteen (8–12)	98.97% (96)	93.75% (135)	
Teenage (13–16)	1.03% (1)	6.25% (9)	
Child gender (% male)	48.45% (47)	54.86% (79)	0.68
**Parent/family variables**			
Parent’s education			1.62
High school diploma or GED	1.03% (1)	0.69% (1)	
Some college or associate’s degree	12.37% (12)	11.81% (17)	
College (bachelor’s) degree	28.87% (28)	29.17% (42)	
Master’s degree	44.33% (43)	38.19% (55)	
Professional (PhD/JD/MD) degree	13.40% (13)	18.75% (27)	
Family income (in thousands)	$130.8 ($75.0)	$169.1 ($114.5)	−3.14**
**School variables**			
School climate	4.16 (0.5)	4.18 (0.46)	–0.37
Public school	75.26% (73)	72.22% (104)	0.14
**Community variables**			
Urbanicity (1 = *large metro* to 6 = *non-core*)	2.04 (1.17)	1.92 (1.12)	0.78
Democratic voting percentage	60.4 (15.64)	64.78 (15.66)	−2.13*

^+^p < 0.10, *p < 0.05, **p < 0.01.

**TABLE 2:** Parent-reported victimization by family type.

	Family type	
	Heterosexual	Same-sex
Variable	% (*n*)	% (*n*)
Threatening to hurt or hit your child	10.31% (10)	13.19% (19)
Pushing or shoving your child	22.68% (22)	13.19% (19)
Hitting, slapping, or kicking your child	9.28% (9)	11.11% (16)
Teasing, picking on, or making fun of your child	34.02% (33)	27.78% (40)
Stealing your child’s things	6.19% (6)	5.56% (8)
Emailing/e-messaging your child or posting something about your child on the internet	0% (0)	0.69% (1)
Spreading rumors or lies about your child	4.12% (4)	4.17% (6)
Ignoring or leaving your child out on purpose	25.77% (25)	19.44% (28)
Making sexual comments or gestures to your child	3.09% (3)	3.47% (5)

### Procedure

Ethical approval for this study was obtained from Clark University’s Institutional Review Board. Participants, all of whom provided informed consent, were assessed 8 years after becoming first-time parents via adoption. Inclusion criteria for the original study were that both partners must be first-time parents, and adopting. Parents were originally recruited from adoption agencies and LGBTQ organizations in the United States for a study of the transition to adoptive parenthood. They participated in several follow-up assessments (e.g., when their children were transitioning to kindergarten). Eight years post-adoption, they were invited to participate in a follow-up online survey focusing on their eldest adopted child’s transition to elementary school; data are drawn from this assessment. Questions about child behavior and experiences focused on the target (i.e., oldest) child. Although 59 families (43% of the current sample) had adopted additional children, these children were not the focus of the study.

Parents were also asked whether they were interested and willing in having the target child be interviewed over the telephone. One of the instruments that was administered to children was the victimization measure. Of the 136 families in the study, 95 (69.9%) agreed to have their child be interviewed (*M* age = 8.82, 56.6% boys). When parents declined their children’s participation, we inquired as to why. Among those declining, reasons given included: parental concerns that interview might upset the child [e.g., by emphasizing difference (25%) or bringing up sensitive topics such as adoption and peer difficulties (4%)]; parents’ sense that the child was too shy (15%) or immature (2%) to fully participate; the child has developmental delays such as autism (13%) or other socioemotional/behavioral issues (12%), or is under a lot of stress (12%); and, the child was too busy (13%) or not interested (13%). Sixteen percent of those who declined provided no reason as to why; 26% gave multiple reasons. There was no statistically significant difference by family type in the number of reasons given, c^2^(1) = 0.158, *p* = 0.691.

### Measures

#### Controls

##### Child variables

Child gender (0 = male, 1 = female) was included as a predictor. Child race (1 = of color, 0 = not of color^2^) and age in years were also included as predictors.

##### Parent variables

Family income in tens of thousands of dollars, and parent education (1−6 scale; 1 = less than high school and 6 = PhD/MD/JD) were entered as continuous predictors.

#### Study Variables

##### Victimization

Parents’ perceptions of peer victimization/bullying (Waasdorp et al., 2011) were obtained using a 9-item scale.^3^ The original items referred to the last month (i.e., “Within the last month, has someone repeatedly tried to hurt your child or make your child feel bad by…”); we altered this to refer to the past year to capture a broader time frame. The response options included five forms of *direct victimization* (i.e., threatened to hurt or hit your child; pushing or shoving your child; hitting, slapping, or kicking your child; teasing, picking on, or making fun of your child; stealing your child’s things) and four forms of *indirect victimization* (i.e., e-mailing/e-messaging your child or posting something online about your child; spreading rumors or lies about your child; ignoring or leaving your child out on purpose; making sexual comments or gestures to your child).^4^ We used the sum of direct and indirect victimization as the outcome. The overall mean for victimization was 1.05 (*SD* = 1.44).

The relationship between parents’ perceptions of victimization experiences is measured by the intraclass correlation (ICC). This dependence in victimization scores requires the use of multilevel modeling (MLM) for analyses predicting victimization. Parents’ reports of victimization were moderately correlated, *ICC* = 0.52 (the ICCs were 0.73, 0.27, and 0.51 for lesbian, gay male, and heterosexual parents respectively), and thus MLM was used.

The subset of children who were asked the same set of questions were presented with the query, “During the past year, has anyone tried to hurt you or make you feel bad by…”. A total of 80 children were interviewed over the phone and responded to these items.

##### Responses to victimization

Parents’ responses to different types of peer victimization were evaluated (Waasdorp et al., 2011). Parents who endorsed at least one type of victimization were asked to indicate which of the following seven actions they had taken in response (yes = 1, no = 0): talk to the bully, talk to the bully’s parents, talk to the child, talk to the child’s teacher, talk to the school counselor, talk to a school administrator, and ignore it/do nothing.

##### Psychological adjustment

Parents completed the Child Behavioral Checklist (CBCL/6-18), which is one of the most widely used measures of children’s behavior and has solid validity and reliability (Achenbach and Rescorla, 2000). Parents rate 112 child behaviors (e.g., “argues a lot”) as 0 (“not true” of the child), 1 (“somewhat or sometimes true”), or 2 (“very true or often true”). Higher scores indicate more problems. The CBCL assesses internalizing behaviors (which reflect mood disturbance, including anxiety, depression, and social withdrawal) and externalizing behaviors (which reflect conflict with others and violation of social norms). Raw scores are summed for each subscale and then transformed into *t*-scores. The standard scores are scaled so that 50 is average for the youth’s age and gender, with a standard deviation of 10 points. Higher scores indicate greater problems. The *ICC* = 0.39 for internalizing (*M* = 50.79, *SD* = 10.54; *ICC*’s were 0.38, 0.36, and 0.25 for lesbian, gay male, and heterosexual parents respectively) and *ICC* = 0.58 for externalizing (*M* = 52.94, *SD* = 10.88; *ICC*’s were 0.84, 0.78, and 0.39 for lesbian, gay male, and heterosexual parents respectively).

##### Family type

A variable indicating whether parents were in a same-sex (1) versus different-sex (0) relationship was included.

##### School climate

We used a four-item measure of school social climate (Schueler et al., 2014). Parents respond to each item using a 5-point scale (1 = not at all, 5 = a tremendous amount). These items were: To what extent do you think that the children at your child’s school enjoy going to school there? Overall, how much respect do you think the children at your child’s school have for the staff? Overall, how much respect do you think the teachers at your child’s school have for the children? How much does the school value the diversity of children’s backgrounds? Cronbach’s alpha for the measure was 0.75.

##### Public vs. private school

We included school type (public = 1; private = 0) as a predictor.

##### Urbanicity

We used participants’ city and state to determine their county of residence, which can be mapped onto United States Census designations for urbanicity. Level of urbanicity, measured (using United States Census designations) as 0 = large central metro, 1 = large fringe metro, 2 = medium metro, 3 = small metro, 4 = micropolitan, and 5 = non-core, was used in the model as a continuous predictor (U.S. Census Bureau, 2013, 2016). Large central metro counties are counties in metropolitan statistical areas (MSA) of one million or more population that (1) contain the entire population of the largest principal city of the MSA, (2) are completely contained in the largest principal city of the MSA, or (3) contain at least 250,000 residents of any principal city of the MSA. Large fringe metro counties are counties in MSA’s of one million or more population that do not qualify as large central. Medium metro counties are counties in MSAs of 250,000 to 999,999 population. Small metro counties are counties in MSAs of less than 250,000 population. Micropolitan counties are counties in micropolitan statistical areas, and non-core counties (i.e., rural) are non-metropolitan counties that are not in a micropolitan statistical area (Centers for Disease Control, 2019).

##### Community political leaning

Percentage of residents in the participants’ county who voted Democrat in the last election was included as a predictor, main effect only.^5^

### Data Analysis

Of the 261 parents who participated in the study, 241 (105 dyads and 31 individuals) had information on the important variables for the current study (e.g., perceptions of the child’s victimization experiences, information about the urbanicity of their residence) and were parents of children aged 17 years old or younger (ultimately, there were no youth older than 16 included in the sample). Of these 241 parents, 65 were gay male parents, 79 were lesbian parents, and 97 were heterosexual parents (a total of 46 men and 51 women representing 136 families).

Generalized Linear Mixed Modeling (GLMM) was used to predict parents’ reports of victimization due to the observations from parents within a family not being independent. Specifically, random intercept models were used to estimate the variance in victimization across families, thus producing correct standard errors. Non-independence in dyadic data is normally modeled as a correlation between partners’ residuals, allowing for the possibility of negative non-independence; however, because there is a positive relationship in parents’ accounts of victimization, random intercept models are appropriate (Kenny et al., 2006). Adding random slopes to dyadic models is generally not advised because there are only two observations per cluster. Further, victimization was recorded as a sum of experiences, with most parents reporting that their children experienced no victimization. This type of data (small counts with a large amount of zeros) is most appropriately modeled with a Poisson distribution allowing for over-dispersion, or the quasi-Poisson^6^ and Penalized Quasi-Likelihood Estimation with the *MASS* package (Version 7.3.51.1; Venables and Ripley, 2002) in R (Version 3.5.2; R Core Team, 2018). All analyses were conducted in R using the R-packages *lme4* (Version 1.1.17; Bates et al., 2015), *lmerTest* (Version 3.0.1; Kuznetsova et al., 2017), *nlme* (Version 3.1.137; Pinheiro et al., 2018) and *psych* (Version 1.8.4; Revelle, 2018). Figures were created with the *ggplot2* (Version 3.1.0; Wickham, 2016) package. For the same reasons, a single-level GLM quasi-Poisson model is also used to model children’s self-reported victimization, but MLM assuming a normal distribution is used when modeling parent-reported child internalizing/externalizing symptoms. More detail is given about these analyses in each of the corresponding sub-sections of the results section.

Key predictors of each outcome variable included family type (same- vs. different-sex^7^), school climate, school type (public vs. private), urbanicity, community political leaning, and the interaction of family type and urbanicity. Control variables included the child’s age, gender, and race (of color vs. not), and the parents’ family income and education.

## Results

### Parents’ Reports of Child Victimization

First, we examined predictors of parents’ reports of child victimization using GLMM. A model with only main effects and then the full model with the exploratory interaction of family type and urbanicity were fit to the data. In the main effects only model, there were no significant effects of family type, *b* = −0.34, *exp(b)* = 0.71, *SE* = 0.26, *p* = 0.183, *95% CI* = [−1.83, 3.05], nor urbanicity, *b* = −0.13, *exp(b)* = 0.88, *SE* = 0.13, *p* = 0.292, *95% CI* = [−0.38, 0.11]. The only significant main effect was school climate, which had a negative relationship with parent-reported victimization, *b* = −0.42, *exp(b)* = 0.65, *SE* = 0.18, *p* = 0.024, *95% CI* = [−0.78, −0.07]: that is, parents who viewed their children’s school climate more positively also tended to report their children as having lower levels of victimization.

**FIGURE 1:**
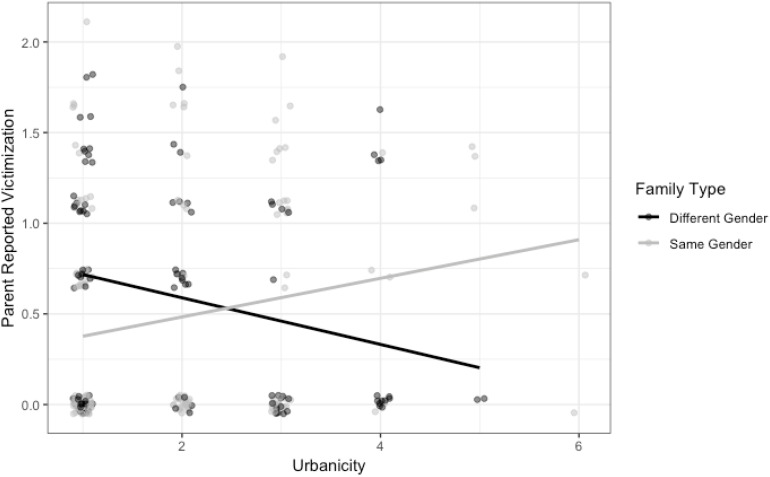
This figure depicts the interaction between urbanicity and family type on parent-reported victimization experiences. Note that points have been jittered horizontally and vertically for visibility purposes in the figure only.

In the full model (Table 3), with all control and predictor variables listed above, there was an interaction of family type (same-sex = 1 versus different-sex = 0) and urbanicity, *b* = 0.47, *exp(b)* = 1.60, *SE* = 0.22, *p* = 0.033, *95% CI* = [0.05, 0.89], such that in large central metro areas, children in LG-parent families were, according to parents, experiencing less victimization than children in heterosexual-parent families, *b* = −0.80, *exp(b)* = 0.45, *SE* = 0.33, *p* = 0.016, *95% CI* = [−1.43, −0.17] (vertical distance between black and gray lines at the far left most point seen in Figure 1)^8^ ; whereas, in non-core (rural) regions, children with LG parents were experiencing more victimization compared to children with heterosexual parents, *b* = 1.09, *exp(b)* = 2.98, *SE* = 0.71, *p* = 0.125, *95% CI* = [−0.27, 2.45] (vertical distance between black and gray lines at the far right most point seen in Figure 1), although this latter simple effect estimate had a large amount of uncertainty as one can see by the relative paucity of data collected from rural areas. In sum, our model predicts that to the extent that a family lives in a more urban community, children with LG parents are, according to parents, victimized less than children with heterosexual parents, and the opposite may be true in more rural areas.^9^ School climate maintained a negative relationship with parent-reported victimization in the full model, *b* = −0.44, *exp(b)* = 0.65, *SE* = 0.18, *p* = 0.020, *95% CI* = [−0.79, −0.08]. No controls or other predictors were significant in the main effects or full models.

**TABLE 3:** Estimates from main effects only and full models.

	Main effects only model					Full model				
Variable	*b*	*Exp(b)*	*df*	*t*	*95% CI*	*b*	*Exp(b)*	*df*	*t*	*95% CI*
Intercept	0.61	1.83	132	0.48	[−1.83, 3.05]	1.09	2.98	132	0.87	[−1.34, 3.52]
Family type	−0.34	0.71	132	−1.34	[−0.84, 0.15]	−0.80	0.45	132	−2.43*	[−1.43, −0.17]
Urbanicity	−0.13	0.88	95	−1.06	[−0.38, 0.11]	−0.42	0.66	94	−2.29*	[−0.78, −0.06]
Percent voting Democratic	0.00	1.00	95	−0.28	[−0.02, 0.01]	0.00	1.00	94	−0.51	[−0.02, 0.01]
Child of color	0.11	1.12	132	0.41	[−0.41, 0.63]	0.06	1.06	132	0.22	[−0.45, 0.57]
Child age	0.13	1.14	132	1.68^+^	[−0.02, 0.28]	0.13	1.14	132	1.71^+^	[−0.02, 0.28]
Gender (male = 1)	0.07	1.07	95	0.30	[−0.39, 0.54]	0.03	1.04	94	0.15	[−0.42, 0.49]
Education	0.01	1.01	95	0.13	[−0.16, 0.19]	0.01	1.01	94	0.09	[−0.17, 0.18]
Family income (in $10k)	−0.01	0.99	95	−0.49	[−0.03, 0.02]	−0.01	0.99	94	−0.36	[−0.03, 0.02]
School social climate	−0.42	0.65	95	−2.30*	[−0.78, −0.07]	−0.44	0.65	94	−2.36*	[−0.79, −0.08]
Public school	0.03	1.03	95	0.11	[−0.46, 0.52]	0.06	1.06	94	0.25	[−0.42, 0.55]
Family type × urbanicity	–	–	–	–	–	0.47	1.6	94	2.16*	[0.05, 0.89]

^+^p < 0.10, *p < 0.05. For family type, LG parent families are coded as 1 and heterosexual parent families are coded as 0. For urbanicity, 0 equals large central metro.

### Exploratory Analysis of Child Victimization Reports

Among those children with child follow-up survey data who were under age 18, there were 67 who reported any victimization experiences and 16 who reported no victimization, while three were missing.^10^ However, of the 241 parents in the analyses reported above, 138 had data from children’s reports of victimization (74 families) and 103 (62 families) were missing child’s reports. It is quite possible that the children who were missing self-reports of victimization were indeed those children who experience more victimization, in which case the data is missing not at random (MNAR) and any interpretations gleaned from the data would be seriously limited. First, before analyzing child victimization reports, in hopes of providing evidence that the data are not MNAR, we assessed whether the missingness on the child’s victimization reports were not associated with the *parents’* reports of victimization by including an indicator of child missingness (missing = 1, not missing = 0) as a predictor of parent-reported victimization in a model by itself alone (*p* = 0.990) and as an addition in the models reported above (all controls included, *p* = 0.937). Parents’ reports of victimization were not associated with whether or not their child participated. Second, to assess if missingness on child victimization reports were associated with any of the study variables (MNAR), we ran a logistic regression model with missingness on victimization as the outcome variable and the following predictors: family type, school climate, school type, urbanicity, community political leaning, child race, child gender, child age, parent education, and family income. Only child gender was significantly associated with missingness *exp(b)* = 2.20, *p* = 0.048, with girls 2.20 times as likely to be missing as boys. We took these two analyses as evidence that the data were missing at random (MAR). The choice was made to simply control for the child’s gender in all analyses reported in the main results section for the analysis of child’s victimization reports, instead of attempting to impute such a large portion of missing data.

In a model including the same predictors and control variables as in the parent-reported victimization models fit above, we tested the relationship between family type, urbanicity, and child-reported victimization (*n* = 80). Children’s reports did not follow a normal distribution, as was the case with parent’s reports; thus, a generalized linear model (single-level GLM) assuming a quasi-Poisson distribution was used. This was a single-level model because we included only one child from each family in the analysis. There were no statistically significant associations with children’s reports of victimization in this full model nor in a model including only family type (*p* = 0.934), urbanicity (*p* = 0.141), and the interaction of the two (*p* = 0.457). See Table 4 for a correlation matrix of child reports of victimization and all study variables. Notably, child reports of victimization were correlated positively with parent reports of victimization, *r* = 0.23, *p* = 0.044, but were uncorrelated with family type, *r* = 0.13, *p* = 0.287.

**TABLE 4:** Correlations among child reports of victimization and study variables (*n* = 73 to 80).

	1	2	3	4	5	6	7	8	9	10	11	12	13
1. Child reports of victimization	1												
2. Parent reports of victimization^*a*^	0.28*	1											
3. Family type (1 = LG)	0.13	−0.20^+^	1										
4. Urbanicity	−0.22^+^	–0.01	–0.05	1									
5. CBCL total score^*a*^	0.29*	0.43***	–0.14	–0.11	1								
6. Percent voting Democrat	0.21^+^	0.01	0.24*	−0.39**	0.06	1							
7. Child of color	–0.16	<0.01	0.14	0.02	–0.08	–0.03	1						
8. Child’s age	–0.03	0.13	–0.08	0.17	–0.06	0.02	0.13	1					
9. Child gender (1 = male)	–0.05	0.09	–0.10	0.03	−0.20^+^	0.15	–0.02	–0.11	1				
10. Parent education^*a*^	0.03	–0.09	0.15	0.03	0.13	–0.05	0.14	–0.10	–0.08	1			
11. Family income	0.05	–0.07	0.24*	–0.04	–0.12	0.37**	0.01	–0.09	0.04	0.23^+^	1		
12. School social climate^*a*^	–0.12	–0.18	0.08	−0.19^+^	−0.32**	0.17	0.16	0.08	–0.12	0.02	0.11	1	
13. Public school	0.03	0.10	–0.10	0.03	0.17	−0.22^+^	–0.08	−0.21^+^	–0.10	–0.06	−0.30*	−0.31**	1

^+^p < 0.10, *p < 0.05, **p < 0.01, ***p < 0.001. ^a^The parents’ data was averaged for this correlation matrix. Correlations are provided for the child data only because a full reporting of relationships was not included in the main text, as it was for the parent data.

### Parents Responses to Victimization

There were various ways that parents could respond to victimization experiences, including to talk to the bully, talk to the bully’s parents, talk to their child, talk to the child’s teacher, talk to the school counselor, talk to the school administrator, and ignore it/do nothing. Parents who indicated that their children had never been victimized were missing all of the responses to victimization variables: they had no victimization to respond to. Among those who endorsed *any* victimization (*n* = 113; 46.9%), we used chi-square tests to assess whether there were differences between family types (same-sex vs. different-sex) in their likelihood of responding in each of the seven ways. Many of the expected cell counts were very small, less than five, and thus *p*-values based on Fisher exact tests are reported. Table 5 presents the *n* and% for each type of response to victimization, for the total sample and by family type.

**TABLE 5:** Percentage and number of parents reporting each type of responses to child’s victimization experiences by family type.

	Family type		Full sample
Response	Heterosexual	Same-sex	
	*n* (%)	*n* (%)	*n* (%)
Talk to the bully	1 (2.27)	7 (14.00)	8 (8.51)
Talk to the bully’s parents	4 (9.09)	6 (11.76)	10 (10.53)
Talk to their child	39 (84.78)	53 (98.15)	92 (92.00)
Talk to the child’s teacher	28 (62.22)	41 (77.36)	69 (70.41)
Talk to the school counselor	11 (25)	18 (35.29)	29 (30.53)
Talk to the school administrator	13 (29.55)	26 (52)	39 (41.49)
Ignore/do nothing	2 (4.55)	4 (8.33)	6 (6.52)

*Parents could have reported more than one type of response or have been missing on any one response (12.39% reported none of the responses, 19.47% one response, 25.66% two responses, 23.89% three responses, 13.27% four responses, 3.54% five responses, and 1.77% of parents reported six responses). Percentages are percentages of non-missing on that response type.*

There was no evidence of an association between family type and talking to the bully’s parents, *p* = 0.748, nor talking to the child’s teacher, *p* = 0.123, nor talking to the school counselor, *p* = 0.372, nor ignoring/doing nothing, *p* = 0.679. There was an association between family type and responding by talking to the bully, *p* = 0.003, with 2.27% (only one) heterosexual parents reporting talking to the bully and 14% of LG parents reporting talking to the bully. There was also an association between family type and talking to their child, *p* = 0.023, with 84.8% of heterosexual parents and 98.2% of LG parents reporting this response; and talking to a school administrator, *p* = 0.036, with only 29.6% of heterosexual parents reporting talking to an administrator and 52.0% of LG parents reporting this response.

### Psychological Adjustment

Next, we explored the relationship between victimization and child adjustment. Given the findings that emerged in predicting victimization, we tested whether family type and urbanicity were related to children’s internalizing and externalizing scores, as reported by parents on the CBCL, and whether there was evidence for mediation of this relationship by victimization. In other words, we wondered whether children with LG parents might experience poorer psychological adjustment than children with heterosexual parents to the extent that they are in more rural areas, and if this relationship could be explained, in part, by increased victimization.

Child Behavioral Checklist *t*-scores for internalizing and externalizing symptoms were normally distributed; thus, the final models reported here assume normality. MLM, with a random intercept model for dyads, was again used due to parents’ reports on the CBCL being dependent. Urbanicity, family type, and the interaction of urbanicity and family type were included as predictors. Predictors and controls were the same as in the model for victimization. Due to complexities resulting from differing distributional assumptions for victimization, the mediator (quasi-Poisson), and the outcome variable (normal), the presence of indirect effects were inferred simply by testing the paths from the family type by urbanicity to victimization and from victimization to CBCL (controlling for the family type by urbanicity interaction), separately.

#### Internalizing

**FIGURE 2:**
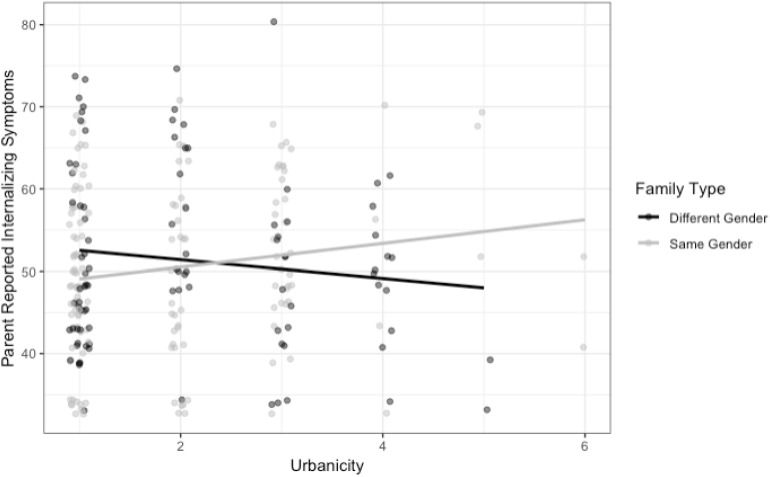
This figure depicts the interaction between urbanicity and family type on parent-reported internalizing symptoms. Note that points have been jittered horizontally and vertically for visibility purposes in the figure only.

As predicted, and consistent with the pattern observed for victimization, there was a significant interaction between family type and urbanicity, *b* = 2.83, *SE* = 1.41, *p* = 0.046, *95% CI* = [0.14, 5.48] (see Figure 2), such that in large central metro areas, children in LG-parent families had fewer internalizing symptoms than children in heterosexual-parent families, *b* = −3.09, *SE* = 2.17, *p* = 0.157, *95% CI* = [−7.18, 1.03], and in non-core (rural) regions, children with LG parents had more symptoms than children with heterosexual parents, *b* = 8.24, *SE* = 4.54, *p* = 0.072, *95% CI* = [−0.44, 16.80]. School climate also had a negative effect on internalizing symptoms, *b* = −5.75, *SE* = 1.43, *p* < 0.001, *95% CI* = [−8.46, −2.95]: Parents who reported less positive school climates reported more child internalizing symptoms. When parent-reported victimization was included in this model, the interaction of urbanicity and family type was reduced slightly and no longer significant, *b* = 2.27, *SE* = 1.39, *p* = 0.106, *95% CI* = [−0.40, 4.88], providing evidence for mediation. As expected, there was a significant positive relationship between victimization and internalizing symptoms, *b* = 1.21, *SE* = 0.50, *p* = 0.017, *95% CI* = [0.26, 2.20]: Children with higher levels of parent-reported victimization also had higher levels of parent-reported internalizing symptoms. No other predictors or controls were significant.

#### Externalizing

In contrast to the results for internalizing symptoms, for externalizing symptoms, there was no interaction of family type and urbanicity, *b* = 1.99, *SE* = 1.49, *p* = 0.183, *95% CI* = [−0.85, 4.81]^11^, although it was in the same direction as above. Only school climate was significantly related to externalizing symptoms, *b* = −6.01, *SE* = 1.35, *p* < 0.001, *95% CI* = [−8.65, −3.43], such that children who attended schools that were rated less positively by parents also had higher levels of parent-reported externalizing symptoms. As with internalizing symptoms, there was a significant positive relationship between parents’ reports of victimization and externalizing symptoms (controlling for family type and urbanicity, and their interaction), *b* = 1.39, *SE* = 0.49, *p* = 0.005, *95% CI* = [0.47, 2.39]. None of the other predictors or controls were significant.

## Discussion

This study contributes to a small but growing literature on victimization experiences of children with LG parents. Consistent with prior work (Wainright and Patterson, 2006; Rivers et al., 2008), there were no differences overall in the level of victimization that children reportedly experienced, by family type. However, our investigation of community level variables that have rarely been explored (Power et al., 2014) suggests that the relationship between family structure and victimization may depend on where families live.

We found that in large cities, children in LG-parent families experienced less victimization than children in heterosexual-parent families, according to parent reports, whereas in more rural regions, children with LG parents experienced more victimization than children with heterosexual parents (albeit non-significantly so). This finding dovetails with work on LGBTQ youth which documented greater victimization among youth living in rural communities (Kosciw et al., 2009) and youth living in self-described hostile and small towns (Paceley et al., 2017). The current study—which included both parents’ reports, used a comparison sample of heterosexual parents, and looked at victimization more broadly—also builds on and echoes Power et al. (2014) study of Australian LGBTQ parents, which found that parents living in less urban areas were more likely to report that their children experienced homophobic bullying or discrimination at school. Parents in rural areas were characterized by less of a sense of “connection” to their communities; they were also less “out” and had less contact with LGBTQ people, compared to parents in more urban areas (Power et al., 2014). Perhaps the finding that we observed reflects the reality that families living in more urban areas are more likely to be connected to a visible LGBTQ community and to have LGBTQ friends and neighbors—and to have access to LGBTQ-affirming community service providers, which are more likely in urban settings (Holman and Oswald, 2011; Oswald and Holman, 2013). Perhaps too, rural families are surrounded by less progressive neighbors and parents—which is supported by the fact that the interaction between family type and community political leaning was in the same direction as the interaction between family type and urbanicity. This finding highlights the need to consider contextual factors, such as geographic location and community climate, in studying psychosocial outcomes within LG-parent families in particular. Of course, given the small number of participants living in rural areas in particular, our findings related to urbanicity must be viewed with caution; more research on LG-parent families in diverse contexts is needed.

Prior work has established the importance of school climate, such that schools characterized by positive teacher-student relationships, respect for students, and respect for diversity tend to have lower rates of bullying (Cook et al., 2010). In turn, consistent with some prior work (Attar-Schwartz, 2009), school climate was related to victimization, such that, across family types, parents who reported more negative climate also reported more victimization. Perhaps there is an unexplored mediator of this relationship. Parents’ impressions of school climate may impact their school involvement, such that parents who feel more positively about their children’s schools engage more directly in volunteering, serving on committees, etc. (Beveridge, 2005). LG parents may be especially motivated to actively engage with school communities to ensure that their children are treated fairly (Goldberg et al., 2017), and such involvement may serve to reduce children’s risk of victimization. Future research can examine this possibility. Future work can also seek to establish whether certain aspects of school climate (e.g., the dimension of ‘respect for diversity’) are differentially related to victimization risk within LG- versus heterosexual parent-families, as well as adopted versus non-adopted children.

Some research has documented associations between victimization and mental health (Cook et al., 2010). Research on victimization and adjustment among children with LG parents has been limited by the absence of heterosexual parent comparison groups (Bos and van Balen, 2008; Bos and Gartrell, 2010) and exclusive focus on homophobic, as opposed to general, victimization (Farr et al., 2016). Yet this work has found evidence that LG parent- and teacher-reported victimization is associated with more problem behavior (Bos and van Balen, 2008; Bos and Gartrell, 2010; Farr et al., 2016) and lower self-esteem (Bos and van Balen, 2008) in children. In the current study, we found that children who had higher levels of parent-reported victimization also had higher levels of parent-reported internalizing and externalizing symptoms, controlling for where they lived and the interaction of family structure and urbanicity. And, following the pattern observed in predicting victimization, we also found that in large urban areas, children with LG parents had fewer internalizing symptoms than children with heterosexual parents, whereas in more rural areas, children with LG parents had slightly more internalizing symptoms than children with heterosexual parents. Victimization only partly mediated the relationship between urbanicity and family structure and internalizing symptoms: Children in LG-parent households who resided in less urban settings were reportedly victimized more, and this partly explained their elevated risk for internalizing symptoms.

Parents who reported less positive school climates also reported more internalizing and externalizing symptoms in their children, consistent with prior work documenting the role of positive school climate in reducing mental health and behavioral issues among students (Hendron and Kearney, 2016). Thus, the importance of school climate to child adjustment appears to extend to adopted children and children with LG parents, although more work is needed to explore how specific features of school climate may impact child adjustment in these families. Bos and Gartrell (2010), for example, found that greater stigmatization was associated with more problem behavior in adolescents with LG parents—but this effect was buffered by the presence of LGBTQ curricula, such that stigmatized youth whose schools taught about LGBTQ people and events were less likely to demonstrate problem behavior than stigmatized youth whose schools lacked LGBTQ-inclusive curricula.

Few notable findings emerged in predicting children’s reports of victimization, likely in part because of the much smaller sample of children who provided data. We documented only modest concordance between parents’ and children’s reports of victimization, echoing prior work showing that there is far from perfect agreement between children and parents regarding whether or not children have been bullied (Holt et al., 2009; Larrañaga et al., 2018), thus underscoring the need for future work on LG-parent and adoptive families to consider child reports of victimization. The fact that so many parents declined their children’s participation is a finding in and of itself. Children’s adoptive status likely conferred on some parents a heightened awareness of how participating in a research study might suggest to children that they were different or unique—an impression that some parents acknowledged wanting to avoid. And, given that children were between 8 and 9 on average, some parents may have felt uneasy about allowing their relatively young children to participate in research (Geller et al., 2003; Hoberman et al., 2013). Parents are less likely to decline participation for older (e.g., teen-aged) children, perhaps in part because parents feel more comfortable allowing their teens, who can better comprehend the risks and benefits of research, to decide whether to participate themselves (Hoberman et al., 2013).

There were few differences in parents’ responses to victimization by family type—although this is in part related to the very low base rates and thus small cell sizes for most types of responses. First, somewhat in contrast with Rivers et al. (2008) finding that youth with LG parents were less likely to report that they would turn to school-based supports, we found that LG parents were more likely to talk to administrators than were heterosexual parents. This difference may in part reflect differences in perspective. LG parents may feel more empowered and/or well-positioned to approach school personnel to advocate for their children than *youth* with LG parents—a stance that may be enhanced by parents’ high levels of education and income, which can represent important sources of social capital, particularly in light of other marginalized status(es) (Goldberg et al., 2018). We also found that among parents who reported victimization, LG parents were more likely to talk to their children about such victimization than heterosexual parents, echoing prior work showing that LG adoptive parents are often highly aware of their children’s potential for victimization surrounding multiple marginalized identities, and may engage in socialization around how to handle and respond to bias (Goldberg and Smith, 2016). Finally, LG parents were more likely to report talking to the bully—which is a concern given evidence that this is an undesired response by youth (Mishna et al., 2006) and may be especially upsetting to youth with LG parents, who may, because of their family structure, realistically fear backlash to this type of intervention. These data are intriguing and highlight the need for qualitative research in this area, to better understand parents’ motivations for this approach, and how they engage in it (e.g., how are parents approaching the perpetrator of victimization?)—as well as the perceived consequences of employing this strategy. Notably, the most frequently endorsed responses—talking to the child, and talking to a teacher—were also the most frequently endorsed responses in Waasdorp et al. (2011). And, parents of victimized children endorsed two responses on average (*M* = 2.24), consistent with Waasdorp et al. (2011)—although notably, our study inquired about the past school year, and Waasdorp et al. (2011) asked about the past month, such that the findings are not directly comparable.

School type was unrelated to child victimization. This is interesting amidst prior work suggesting that attending private school may be associated with lower levels of victimization (Brinig and Garnett, 2012; Henkel and Slate, 2013). Given the high levels of education and income among the parents in the sample as a whole, perhaps those who sent their children to public school did so because these were at least moderately safe and/or high in quality, and thus not appreciably different than the private schools that other children in the sample attended. Likewise, the main effect of community political leaning was not significant. Yet the interaction with family type—when tested alone—followed the same pattern as urbanicity, highlighting the interconnectedness of community political leaning and urbanicity, as well as the significance of community context to victimization experiences of children with same-sex parents.

### Limitations and Future Directions

A major limitation of this study is that the data are cross-sectional. Future longitudinal research should seek to determine whether the associations we documented hold up over time. Another major limitation is that we did not include teacher reports and we only had data on child reports from a subset of families. Undoubtedly, the study would be enhanced by the inclusion of both child and teacher reports. In studies of elementary school students, both parents (Holt et al., 2009; Rupp et al., 2018) and teachers (Rupp et al., 2018) report a lower incidence of victimization/bullying than youth themselves. Further, some work suggests that at least some children with LG parents may avoid telling their parents about bullying they experience at school, especially if it is related to parental sexual orientation (Goldberg, 2007, 2010). Thus, children’s reports do represent a unique, important perspective that could be expected to deviate in meaningful ways from parent reports, under some conditions. Furthermore, research that obtains reports of victimization from multiple informants (teachers, peers, self, parents) may enhance prediction of some youth outcomes (Wienke Totura et al., 2009). For example, in one study, higher teacher-youth concordance about victimization was associated with youth academic issues, whereas lower levels were associated with youth moodiness (Wienke Totura et al., 2009).

Given that we relied on parent reports for our main analyses, we have no way of knowing whether, for example, the associations between victimization and child problems might reflect reporting bias. That is, parents with a more negative outlook may have tended to report more negative outcomes in both domains, and, likewise parents with a more positive outlook may have provided more positive assessments of both. Another limitation relates to our modification of Waasdorp et al. (2011) measure of victimization to reflect the past school year. Because of this, our findings are not directly comparable to other studies that inquired about the past month.

We also did not find statistically significant differences between gay father and lesbian mother families. Future work with larger samples should explore whether risk for or processes related to victimization differ for children in gay father versus lesbian mother families. Attitudes toward sexual minority men tend to be more negative than attitudes toward sexual minority women (Costa and Davies, 2012); likewise, attitudes toward gay fathers are more negative than attitudes toward lesbian mothers (Gato and Fontaine, 2016; Webb et al., 2017), whereby, for example, children are believed to be at greater risk for non-normative sexuality development in gay-father households as compared to lesbian-mother households (Gato and Fontaine, 2016). In turn, children with gay fathers may be more vulnerable to peer victimization. Because of the simplistic nature of our child race variable, future work should seek to explore how victimization experiences might vary based on specific racial/ethnic categories. For example, due to the specificity of stigmas and stereotypes related to race and sexuality, a Black male child with two White gay fathers might have a different experience than an Asian male child with two White gay fathers; or a, Black male child with two White lesbian mothers.

Children’s psychological adjustment may also be influenced by a variety of factors that we did not assess in the current study. All of the children in the study were adopted, and prior work has documented associations between pre-adoptive history (including adverse experiences and age of the child at placement) and psychological adjustment (Jones and Morris, 2012). Likewise, post-placement adoption-related processes, such as parents’ level of preparation for the adoption (Goldberg and Smith, 2013) and level of communication within the family about the adoption (Brodzinsky, 2006), have also been linked to children’s adjustment.

Future work should explore resiliency factors that might mediate the association between victimization and well-being, such as strong LG parent-child relationships (Bos and Gartrell, 2010; van Gelderen et al., 2012), peer relationships (van Gelderen et al., 2012), and contact with other children with LG parents (Bos and van Balen, 2008). Indeed, future work should assess not only risk factors but protective factors for victimization in LG-parent families.

Finally, qualitative work that examines experiences of victimization among youth with LG parents is needed. Children of LG parents face a distinct set of stereotypes and assumptions surrounding their parents’ sexuality and its supposed impact on them. For example, Clarke et al. (2004) point out that the issue of homophobic bullying is frequently used to undermine LG-parent families. Children are deemed to be “at risk” for bullying related to their parents’ sexuality, and this is in turn used as a justification for why LGBTQ people should not be parents. Caught in a “web of accountability” (Clarke et al., 2004, p. 531), children may minimize the bullying that is perpetrated upon them, in part to protect their parents and families (Goldberg, 2007). Research that aims to illuminate not only how children with LG parents experience victimization, but how they balance concerns about their family’s image and safety in sharing information about victimization with others (e.g., therapists and school staff), is needed.

## Conclusion

Lesbian and gay parents and their children live throughout the United States and beyond, in communities that vary in their urbanicity, dominant political orientation, and numerous other factors. This study highlights the importance of attending to the proximal and distal contexts, including school, community, state, and national domains, that shape the lives of LG-parent families. Even more specifically, the findings of this study suggest that urbanicity may be an important community feature that directly and indirectly impacts youth and parents through its effects on schools (e.g., via trickle down of community attitudes, values, and practices; Bronfenbrenner, 1988). Living in an area that is less urban—and, in turn, contains fewer LGBTQ residents and services for those residents—may present a risk factor for LG-parent families, a possibility that is supported by prior work as well and deserves more attention by researchers, policy advocates, and school professionals. Yet much more work is needed to understand how LG-parent families and their children in diverse community and school settings seek to prevent and intervene with regards to victimization. Additionally, research is needed that explores the experiences of educators in different regions and settings with regard to LG-parent families, including their differing training needs, in order to best understand and support these families.

Educators and practitioners who seek to support LG-parent families and adoptive families must recognize the importance of school and community context in shaping these families’ vulnerabilities and resiliencies. They should consider how state, community, and school politics and policies may impact children with LG parents in subtle ways that are difficult to discern (e.g., via the impact of climate, or the availability of LG parent-family inclusive resources) as well as in settings that are rarely considered (e.g., the bus stop; the cafeteria; recess). Educators and practitioners working in less urban areas in particular should carefully evaluate the ways in which diverse families and children may be implicitly excluded and victimized. Finally, all educators and family practitioners should seek ways to engage in social and political advocacy on behalf of diverse and potentially marginalized families, including LG-parent families.

## Data Availability Statement

The datasets generated for this study are available on request to the corresponding author.

## Ethics Statement

The studies involving human participants were reviewed and approved by Clark University. The patients/participants provided their written informed consent to participate in this study.

## Author Contributions

AG wrote the literature review, methods, portions of the results, and discussion. RG wrote the majority of the results, and portions of the methods and discussion.

## Conflict of Interest

The authors declare that the research was conducted in the absence of any commercial or financial relationships that could be construed as a potential conflict of interest.

## Footnotes

1 LG, or lesbian/gay-parent families, refers to families headed by female or male couples respectively. Not all individuals in these couples identified as lesbian/gay (a minority identified as bisexual or queer), but we refer to them as LG for brevity, whereby family structure as opposed to individual sexual identity is emphasized.

2 Children who were not White (i.e., children who were Latina/o/x, African American, Asian, or were biracial or multiracial) were categorized as children of color. The term “person of color” is primarily used in the United States and Canada to describe any person who is not considered White.

3 One item was dropped (“called your child bad words”) from the 10-item scale, as pilot participants viewed this item as redundant with “teasing, picking on, or making fun of your child.”

4 Due to the small overall number of victimization experiences (see Table 2), the low Cronbach’s alpha for indirect victimization (0.34, compared to 0.69 for direct), and because the direction of key results was the same across the two forms of victimization, the sum of direct and indirect victimization was used as the outcome (Cronbach’s alpha for all 9-items = 0.68). Of note is that although we report the alphas for the interested reader, we do not expect these items to measure a singular construct and/or correlate to one another.

5 Urbanicity and community political leaning were moderately negatively correlated in this sample, *r* = −0.46, *p* < 0.001, as would be expected. Due to this association and the multicollinearity it introduces into the model, when the urbanicity × family type and political leaning × family type interactions are both included in the model, neither interaction is significant, but both are significant on their own. The choice was made to include only the urbanicity × family type interaction in the final model, in part because Power et al. (2014) examined urbanicity specifically. The main effect of political leaning is still included, and thus the effects of urbanicity reported in the main text are *controlling* for the percentage of the community voting Democratic.

6 There are virtually no changes to the results when using a Poisson distribution instead of a quasi-Poisson distribution, aside from the loss of the dispersion parameter. The choice was made to use the quasi-Poisson due to the differences between the estimates for the SD (= 1.44) and mean (= 1.05) of victimization. Further, performing a test for overdispersion also indicates that this parameter is needed (Cameron and Trivedi, 1990).

7 Models treating the family type variable as a three-level (lesbian, gay male, heterosexual) categorical variable were also fit to the data. There were no differences in victimization or the effect of urbanicity between lesbian and gay male parent groups, and further, the effects for these two groups were in the same direction and of similar size; thus, it was decided that models defining family type as same-sex versus heterosexual were adequate.

8 The interaction between family type and urbanicity remains statistically significant after removing the few cases of same-sex parent families in rural (non-core) areas, *p* = 0.043.

9 The significant interaction of community political leaning × family type when included in the model without the urbanicity × family type interaction is such that heterosexual parents report more victimization of their children than same-sex parents in more progressive communities while same-sex parents report more victimization than heterosexual parents in more conservative communities (*b* = −0.03, *SE* = 0.02, *p* = 0.026).

10 The number of children with usable data was reduced to 80, because three were missing data on victimization, and three were excluded because they had a sibling (adopted at the same time) who (a) completed the victimization measure, and (b) was the child about whom the parents completed the victimization measure.

11 Sensitivity analysis confirmed that the interaction between family type and urbanicity remained statistically significant for internalizing symptoms (*p* = 0.013), and is statistically significant for externalizing symptoms (*p* = 0.028), after removing the cases of same-sex parent families in rural areas.
